# The effects of task difficulty, novelty and the size of the search space on intrinsically motivated exploration

**DOI:** 10.3389/fnins.2014.00317

**Published:** 2014-10-14

**Authors:** Adrien F. Baranes, Pierre-Yves Oudeyer, Jacqueline Gottlieb

**Affiliations:** ^1^Department of Neuroscience, Columbia University Medical CenterNew York, NY, USA; ^2^InriaBordeaux, France; ^3^Ensta ParisTechParis, France; ^4^Kavli Institute for Brain Science, Columbia UniversityNew York, NY, USA

**Keywords:** intrinsic motivation, decision making, exploration, novelty, video games

## Abstract

Devising efficient strategies for exploration in large open-ended spaces is one of the most difficult computational problems of intelligent organisms. Because the available rewards are ambiguous or unknown during the exploratory phase, subjects must act in intrinsically motivated fashion. However, a vast majority of behavioral and neural studies to date have focused on decision making in reward-based tasks, and the rules guiding intrinsically motivated exploration remain largely unknown. To examine this question we developed a paradigm for systematically testing the choices of human observers in a free play context. Adult subjects played a series of short computer games of variable difficulty, and freely choose which game they wished to sample without external guidance or physical rewards. Subjects performed the task in three distinct conditions where they sampled from a small or a large choice set (7 vs. 64 possible levels of difficulty), and where they did or did not have the possibility to sample new games at a constant level of difficulty. We show that despite the absence of external constraints, the subjects spontaneously adopted a structured exploration strategy whereby they (1) started with easier games and progressed to more difficult games, (2) sampled the entire choice set including extremely difficult games that could not be learnt, (3) repeated moderately and high difficulty games much more frequently than was predicted by chance, and (4) had higher repetition rates and chose higher speeds if they could generate new sequences at a constant level of difficulty. The results suggest that intrinsically motivated exploration is shaped by several factors including task difficulty, novelty and the size of the choice set, and these come into play to serve two internal goals—maximize the subjects' knowledge of the available tasks (exploring the limits of the task set), and maximize their competence (performance and skills) across the task set.

## Introduction

Common experience shows that people voluntarily take on new challenges without the benefits of external rewards (e.g., money), suggesting that they are intrinsically motivated to learn and master new tasks. Intrinsic motivation is arguably an important engine behind human creativity and success in development and adult life (Csikszentmihalyi, 1997; Ryan and Deci, 2000; Deci et al., 2001), but it remains poorly understood. Important questions remain about its fundamental mechanisms, including the factors that give rise to intrinsic motivation and the roles that it plays in behavior.

Among the most influential theories of intrinsic motivation is the principle of optimal challenge—also referred to as the autotelic principle (Steels, 2004)—which states that people avoid activities that are too easy or too difficult (and produce, respectively, boredom and frustration), and instead focus on activities with an intermediate level of challenge (Berlyne, 1960; Csikszentmihalyi, 1997). Engagement in optimally challenging tasks can at times induce a highly pleasurable state of “flow,” characterized by feelings of being relaxed, absorbed and in control (Keller and Bless, 2008; Abuhamdeh and Csikszentmihalyi, 2012), suggesting that it triggers internal rewards. This idea is consistent with evidence that the neural networks recruited during intrinsic motivation include subcortical dopamine-recipient structures that process primary (extrinsic) rewards (Kang et al., 2009; Murayama et al., 2010, 2013; Lee et al., 2012; Satterthwaite et al., 2012; Schouppe et al., 2014; Ulrich et al., 2014).

A primary function of systems of intrinsic motivation is thought to be guiding task selection in conditions where external rewards are absent or unknown, such as during exploration. Human beings, along with other intelligent animals, are remarkable in their drive to actively explore their environment, whether in adult activities such as scientific research, or during the extended period of growth and development (Ryan and Deci, 2000; Deci et al., 2001; Gottlieb et al., 2013, Mirolli and Baldassarre, 2013; Gweon et al., 2014; Taffoni et al., 2014; Oudeyer and Smith, in press). Achieving efficient exploration in such open-ended conditions poses significant computational challenges, which stem from the fact that the agent explores in conditions of limited knowledge and time, the fact that it is faced with vast numbers of possible tasks, and the fact that many of these tasks are random or unlearnable and would optimally be avoided (e.g., one would ideally not spend much effort in trying to predict the stock market from the traffic pattern). To explore efficiently under these conditions (i.e., in a way that increases knowledge), agents may rely in part on low level heuristics such as novelty bias or random action selection, but also require systems of intrinsic motivation that assign value to learnable tasks (Schmidhuber, 2006; Oudeyer and Kaplan, 2007; Oudeyer et al., 2007; Lopes and Oudeyer, 2012; Gottlieb et al., 2013, Mirolli and Baldassarre, 2013).

However, beyond these theoretical considerations, our understanding of intrinsically motivated exploration is limited by a paucity of empirical work. Among the rare laboratory studies relevant to this question, Kidd et al. showed that attentional resources can be targeted to visual stimuli of intermediate complexity in free viewing contexts (Kidd et al., 2012), and Taffoni et al. showed that free play in children can be influenced by novelty and the learning of action-outcome contingencies (Taffoni et al., 2014). However, no study has examined the question of how adult humans spontaneously organize their exploration in a free play context, and how these choices depend on fundamental factors such as the number, difficulty and novelty of the available tasks.

Here we began to examine these questions using a new paradigm where human observers played a series of ~70 short computer games (lasting 5–30 s each), and were allowed to choose freely the difficulty of the games that they wished to sample. Our focus was on the ways in which the subjects organized their exploration when presented with small or large choice sets—containing 7 or 64 games of variable difficulty—and when they did or did not have the option to generate new games at a constant difficulty. We show that, despite the absence of external constraints, subjects adopted consistent exploration strategies characterized by several features. Subjects started with easier games and progressed to more difficult games, tended to explore the entire choice set (including extremely difficult games that could not be learnt), repeated moderately and high difficulty games much more frequently than was predicted by chance, and had even higher repetition rates if they could generate new games at a constant level of difficulty. The results are consistent with theoretical predictions that intrinsically motivated exploration is shaped by several factors including task difficulty, novelty and the size of the choice set. They suggest an underlying model whereby the subjects attempt to maximize their knowledge of the available tasks (by exploring the limits of the choice set), as well as their competence (performance and skills) across the range of these tasks.

## Methods

We tested a total of 52 subjects (29 women), who were recruited from the Columbia University community and were compensated for their participation at the rate of $12 per hour. All methods were approved by the Institutional Review Board of the New York State Psychiatric Institute.

### Task

**Figure 1:**
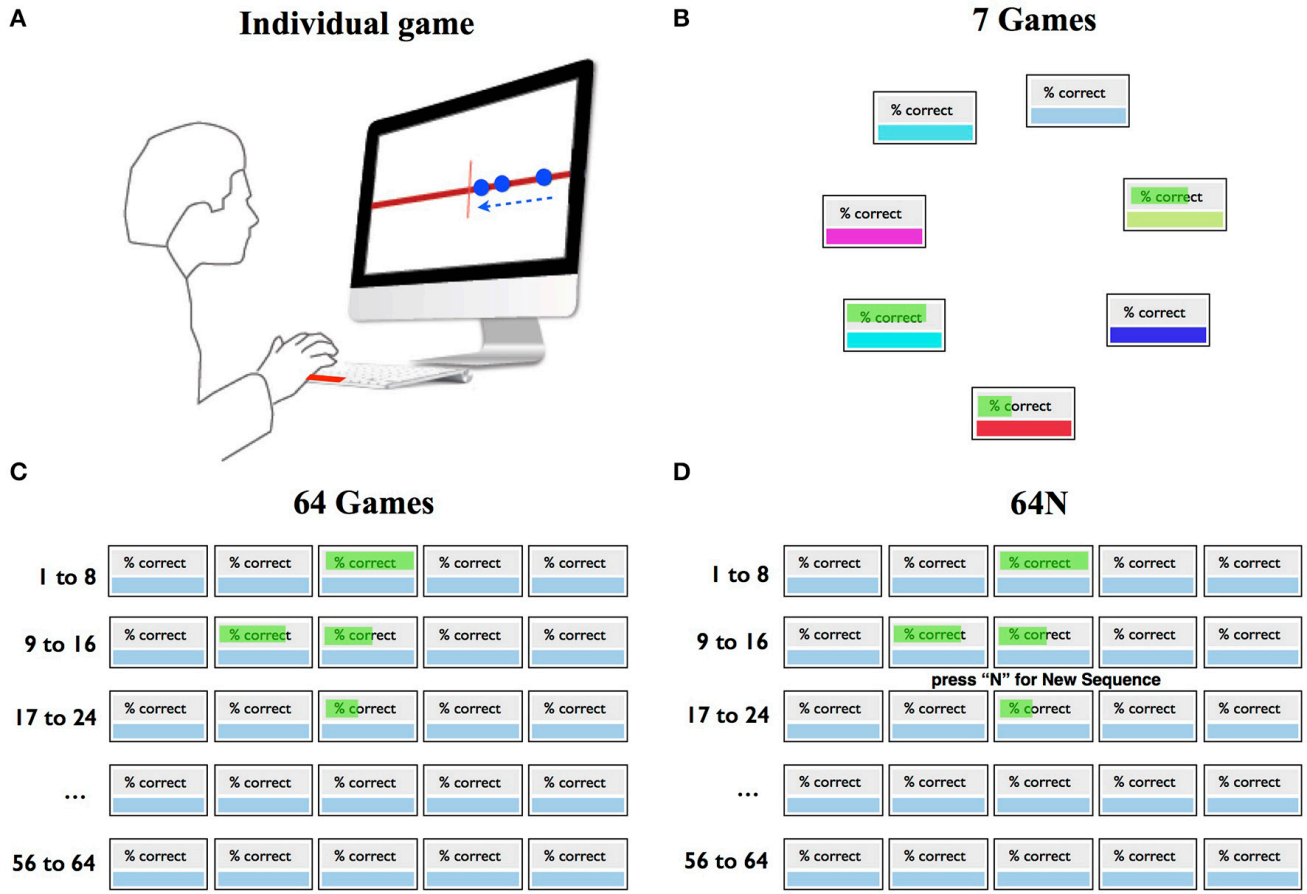
**Task design**. **(A)** Individual game. The subjects pressed a key to intercept a stream of moving dots (arrow) as they crossed the screen center. **(B–D)** Selection screens in the 7-game, 64-game, and 64N-game versions.

Subjects were comfortably seated in front of a computer screen and played a series of simple games freely chosen during the course of a session. In each game the subjects saw a stream of dots that moved from right to left and pressed the space bar to intercept each dot as it crossed a vertical line at the screen center (Figure 1A). Each game consisted of 25 dots that were positioned at variable inter-dot spacings (selected randomly with uniform probability among the values of 1.78°, 3.56°, 7.1°, and 10°) and moved at a constant speed (1°/s–75°/s across different task versions). A game lasted between 5 and 30 s depending on dot speed. Each dot was 0.5° in diameter, and the entire sequence spanned 33° horizontal distance (the right half of the screen). (We give the distances in degrees of visual angle based on an eye-to-screen distance of 50 cm that corresponded to the subjects' approximate position; however, the subjects' heads and bodies were not restrained during the task.)

The key variable of interest was how the subjects chose which game to play as a function of difficulty (speed) and the properties of the choice set. Three groups of subjects experienced 3 choice sets as illustrated in Figure 1, and could freely choose which game to sample. In the 7-game version, the subjects could choose among 7 games represented by distinctly colored boxes randomly arranged in a circular array (Figure 1B). In the 64-game (Figure 1C) and 64N-game versions (Figure 1D), they chose among 64 games arranged in a rectangular array. Each game in the 7 and 64-game versions was characterized by a unique dot sequence and speed. In the 7-game version, game speed was arranged randomly in the choice array, whereas in the 64-game version it increased monotonically along the rows from the top left to the bottom right corners. The 64N version was identical to the 64-game version except that the subjects were given an additional option to request a new sequence at a given speed. Thus, after the subjects selected a box indicating speed, they were prompted to press “N” if they wanted to play a new sequence (Figure 1D), or to press return if they wanted to repeat the same sequence at that speed. If the subjects pressed “N,” a new sequence was generated by randomly shuffling the inter-dot spacings at the requested speed.

After selecting and playing a game, the subjects received feedback indicating their % correct, defined as the fraction of dots that they had successfully intercepted in the preceding game. The feedback was shown graphically through the length of a green horizontal bar superimposed on the selected game (Figures 1B,C). The feedback remained visible on the choice screen throughout the session and was updated only if the subject repeated the game with the performance value for the immediately preceding game.

### Instructions and procedure

At the start of each session the subjects were given a verbal and written explanation of the task and were allowed to play as many practice games as they felt they need (typically, 1 or 2). Thereafter, the subjects were instructed that their only requirement was to play a minimum of 70 games and a minimum of 20 min. This dual requirement was meant to prevent a strategy of simply minimizing time on the task by selecting only the shortest games. Beyond these basic requirements, there were no additional constraints, and the instructions emphasized that the payment for the session was fixed and entirely independent of the game performance or the chosen games.

At the end of the sessions testing the 64-game version we conducted an additional procedure, administered without warning, to determine whether the subjects monitored their progress in the task. After a subject completed the session, we selected 5 games that the subject had played at least twice and which spanned the range of difficulties that he/she had sampled. We asked the subject to play each game once more and then asked him/her to rate (1) how much they estimate that their performance *changed* over the repetitions of the game, and (2) how much do they believe they could improve if they had five more tries. In each case the subjects gave their rating on a scale ranging from −5 (a large decrease in performance) to +5 (a large improvement in performance).

### Data analysis

**Figure 2:**
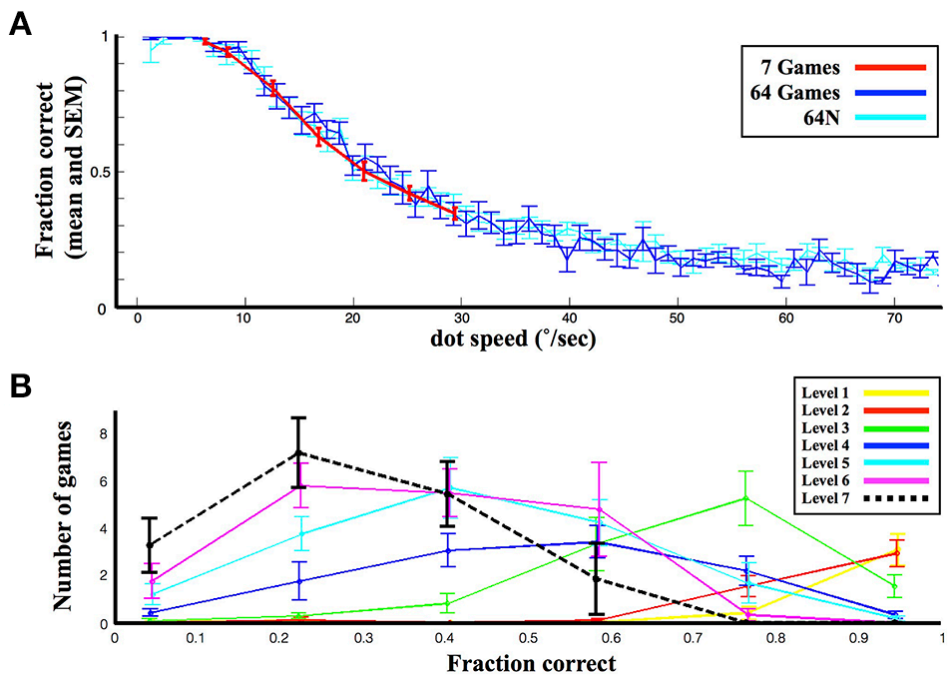
**General performance. (A)** Performance as a function of speed in the 3 versions. Each bin represents the average and standard error (s.e.m.) of the fraction correct for the corresponding dot speed across all the subjects tested. **(B)** The distribution of performance levels in the 7-game condition. The points show the average and s.e.m. (across subjects) of the number of games in each of 6 performance bins.

**Figure 3:**
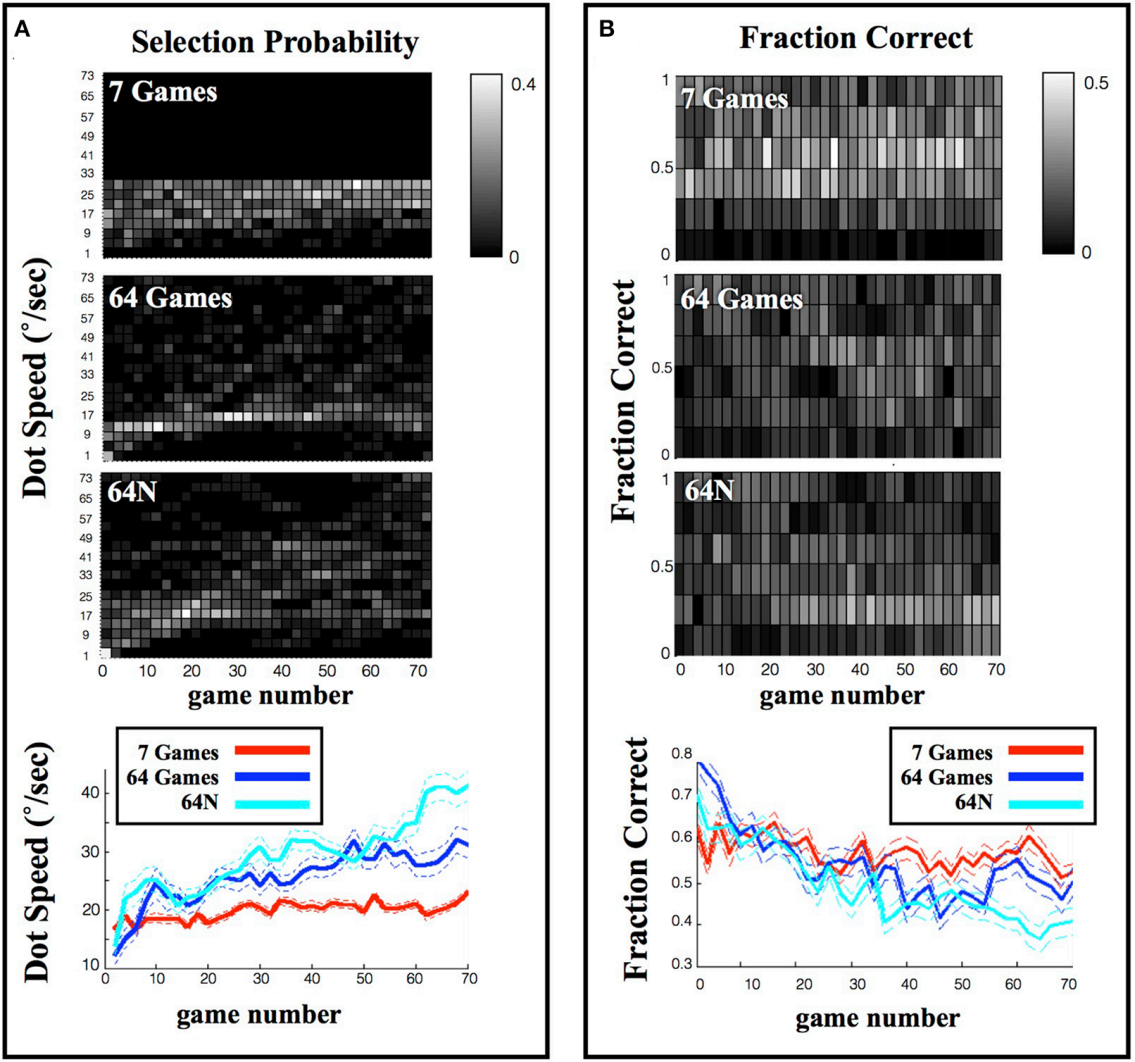
**Selection and performance in the 3 task versions. (A)** Evolution of the selected speed during a session. Each colormap indicates the probability of selection of a given speed, measured across all subjects in a sliding window over the session. The bottom panel shows the average dot speed in each time bin (average and s.e.m. from the corresponding colormaps). **(B)** Evolution of performance during a session. Same as in **(A)**, except that the grayscale indicates the probability of playing at a given fraction correct in each time bin.

**Figure 4:**
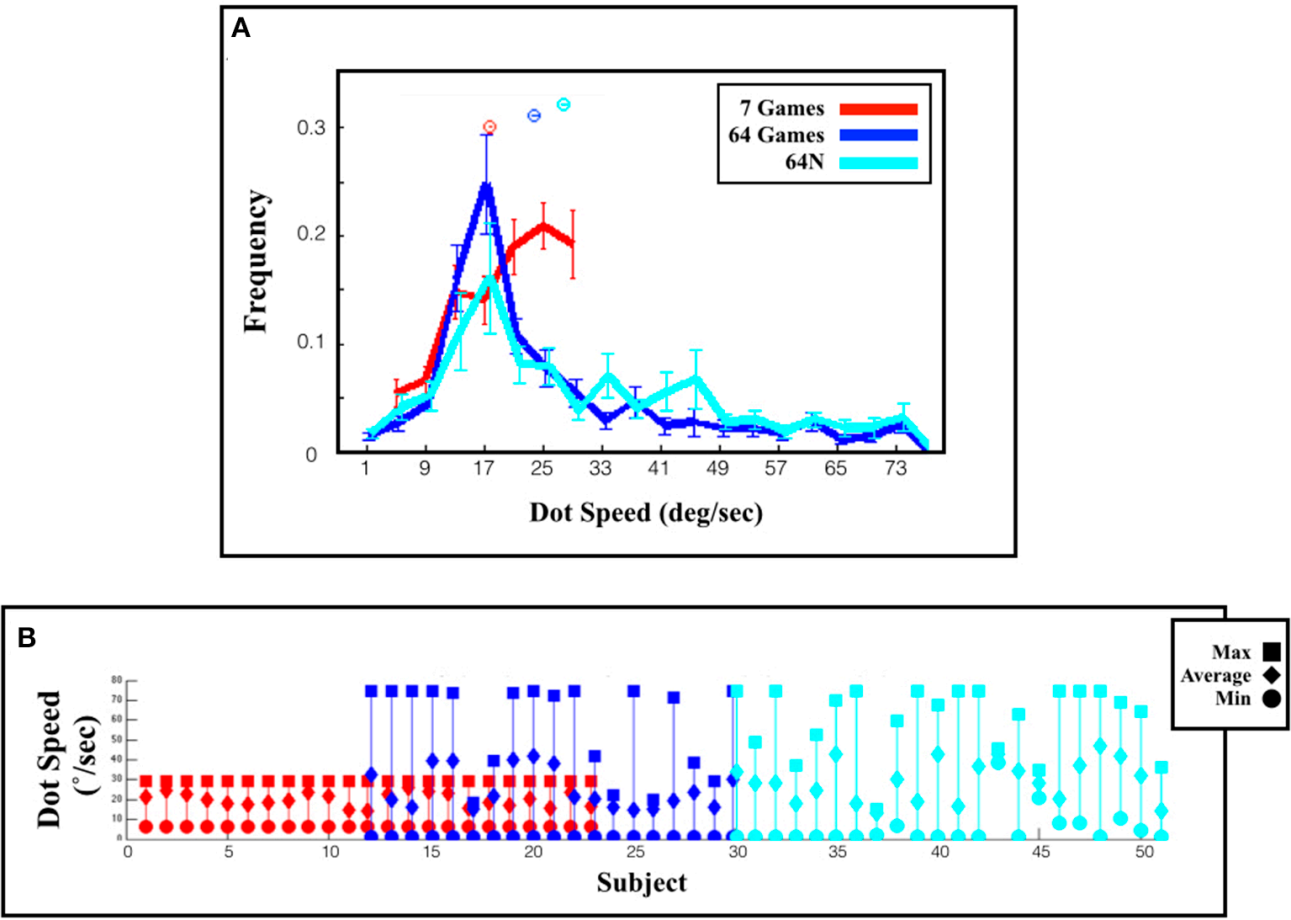
**Range of selected games. (A)** Distribution of the selected speeds (top) and fraction correct (bottom) across an entire session. The values show the mean and s.e.m. across subjects. **(B)** Choices of individual subjects. Each line represents one subject and shows the maximum, minimum and average dot speed selected by that subject. Subjects are ordered according to the task version (or task combination) that they performed, and in chronological order within a task group.

**Figure 5:**
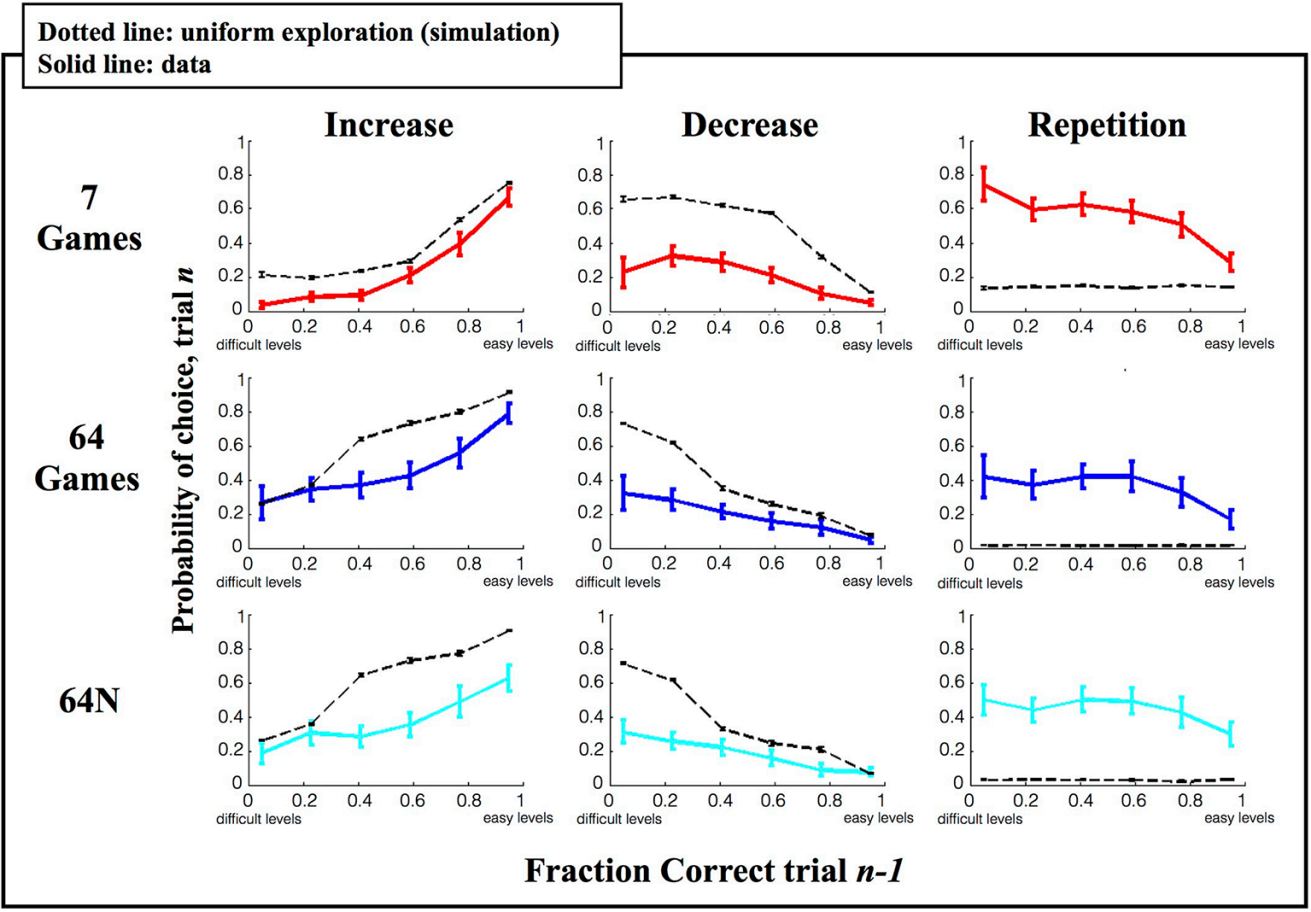
**Local strategy for game selection**. Each point shows the average and s.e.m. of the probability to repeat, increase or decrease difficulty as a function of prior game performance. Solid colored traces show the empirical data, dotted black traces show the results of simulations using a random game selection strategy.

**Figure 6:**
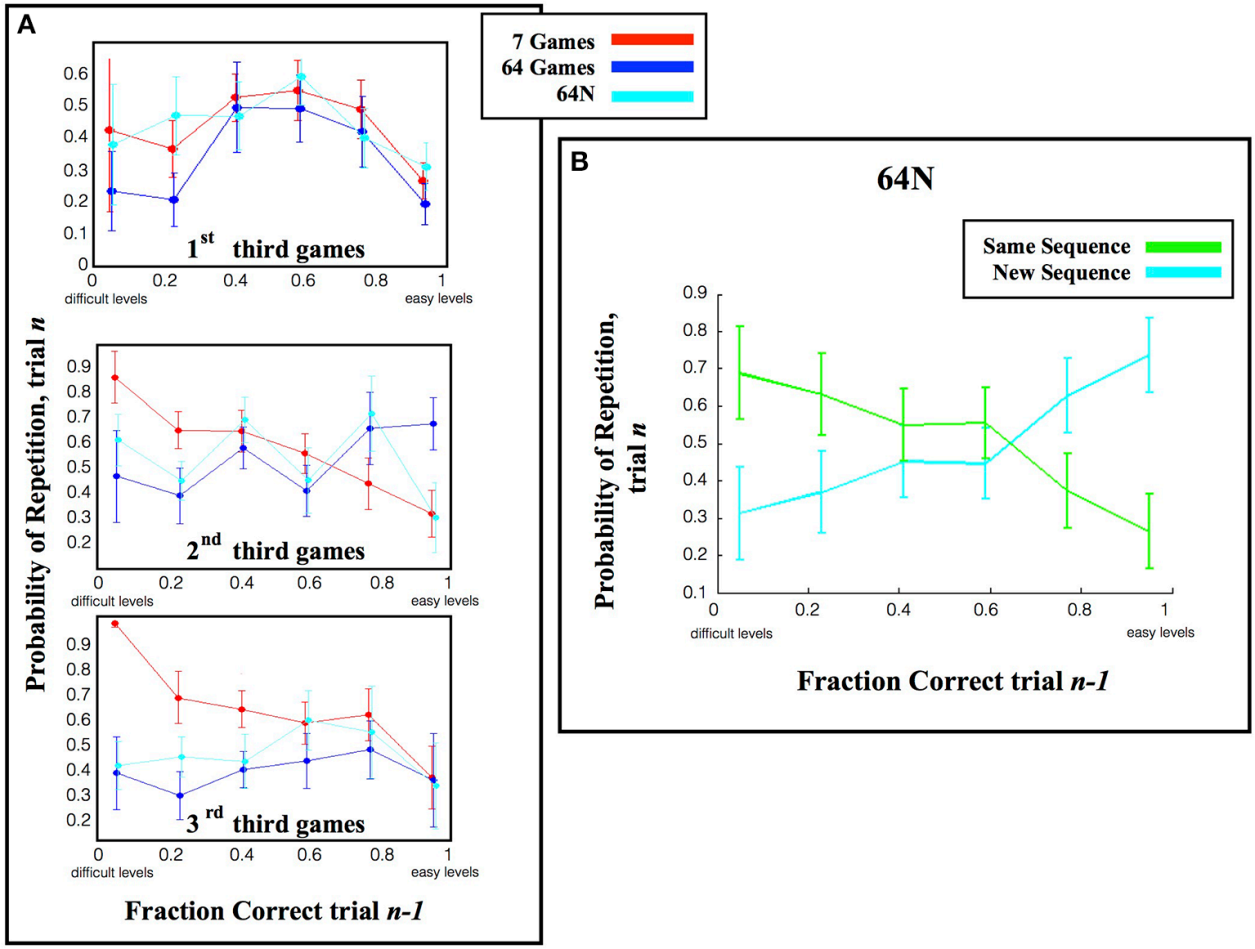
**Repetition rates. (A)** The likelihood of repeating a speed calculated as in Figure 5, but only for the first 1/3 of games in each session. **(B)** The fraction of repetitions, in the 64N versions, where subjects requested the same or a novel sequence. The points show the mean and s.e.m., across subjects, of the tendency to choose a new or familiar sequence when repeating a game of given speed.

**Figure 7:**
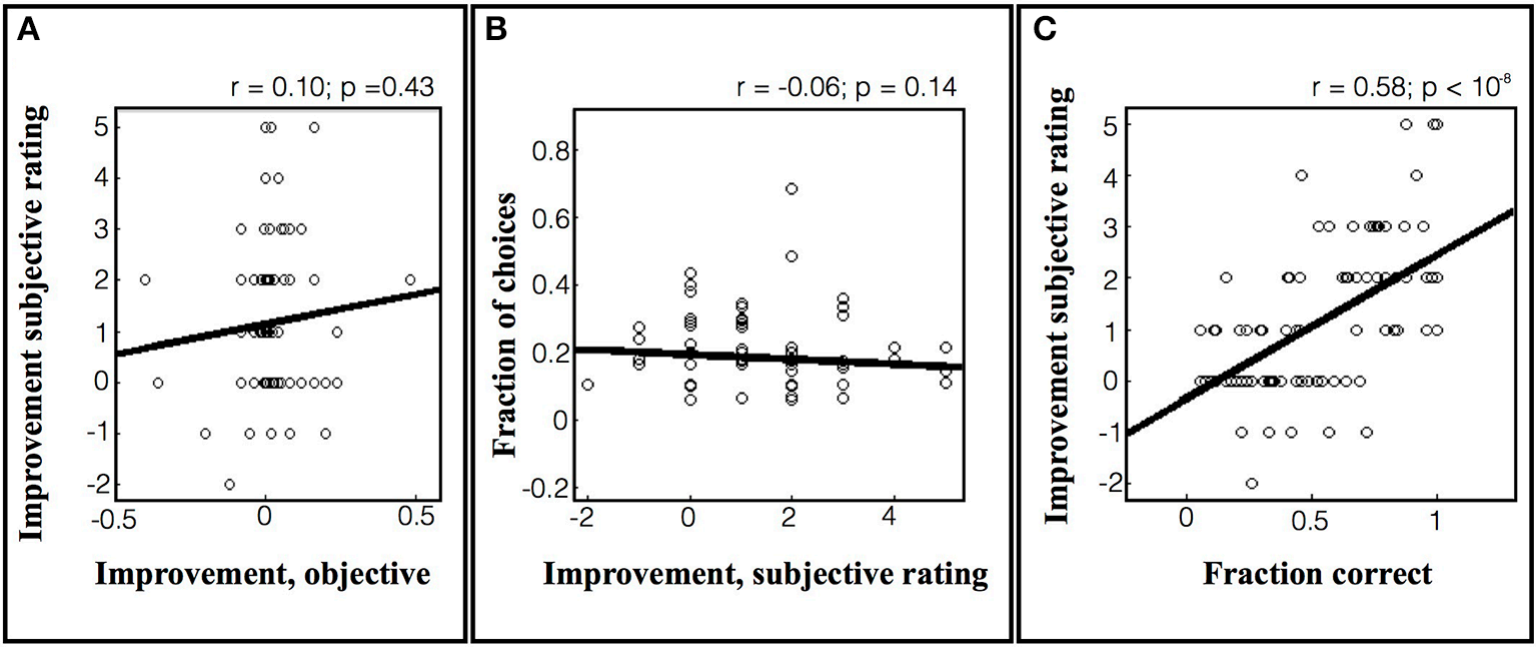
**Subjective rating of learning progress in the 64-game task**. **(A)** Subjective improvement rating as a function of actual improvement. Each point represents a game that was rated by a subject after the end of the session, and the data are pooled across subjects. The y axis shows the subject's rating of his/her own improvement and the x axis shows the objective improvement in units of %correct/game. The lines show best fit linear regression across subjects. **(B)** The probability of selecting a game as a function of the subjective improvement rating. **(C)** Subjective rating as a function of average performance.

For the analyses in Figures 1–**6** the unit of analysis was one subject; we obtained the appropriate measure for one subject and then pooled across the sample. To generate the colormaps in **Figures 3A,B**, we divided each subject's first 70 games into a sliding window of 2 games stepped by 1 game throughout the session, computed the subject's distribution of selected speeds and fraction correct in each bin, and then computed the averages across subjects. To examine the performance-dependent choice strategy (**Figure 5**), we assigned each game that a subject played to one of 6 performance bins (e.g., fractions correct of 0–0.167, 0.168–0.33, etc.), computed the fraction of the following games that were an increase, repeat or decrease in dot speed relative to the previous game, and finally computed the average and standard error of the mean (s.e.m.) across subjects. For the simulations (dotted lines in **Figure 5**), we simulated a set of 300 subjects who selected the game difficulty randomly on each trial. After the virtual subject chose a game, that game was assigned a performance (fraction correct) that was randomly selected (with replacement) from the set of values that were generated by the real subjects for the corresponding dot speed. We then computed the selection rate per subject and mean and s.e.m. across the simulated subjects, as for the real data set. In the analysis of subjective ratings (**Figure 7**) the unit of analysis is one game. For each of the 5 games tested for each subject, we measured the objective improvement (the slope of a linear regression of the % correct across game repetitions), and pooled the data across all subjects and games.

## Results

### General performance

We describe the data from 23, 19, and 22 subjects who completed, respectively, the 7-game, 64-game, and 64N versions. Most subjects completed only one version, while 12 subjects completed the 7 and the 64-game versions, and one subject completed the 64 and 64N versions. We discarded the data from a single subject who selected a single (fast) dot speed throughout the entire session, suggesting that he was trying to minimize time on the task. For the remaining subjects, we verified that they followed the dot speed rather than indiscriminately pressing the bar by comparing the average rate of key presses to the average rate of dot crossings across games of moderate speed that could be reasonably followed (<45°/s). The two rates were equivalent and highly correlated (linear regression slope 0.896 ± 0.05 (average and s.e.m.), *p* < 0.05 in 93% of subjects), showing that the subjects adjusted their presses to the sequence of dots.

As shown in Figure 2A, the 7-game version afforded fewer options and a smaller range of dot speeds relative to the 64- and 64N- versions (6°/s–29°/s vs. 1°/s–75°/s). However, games of equivalent speed elicited equivalent performance in the three versions (*p* = 0.72 for effect of task version, *p* < 0.05 for effect of speed, Two-Way ANOVA for speeds of 6°/s–29°/s). In addition, the average fraction correct on the 7-game version did not fall below 0.4, but the games that the subjects played in this condition spanned the entire performance range (Figure 2B). Thus, the subjects' abilities and performance ranges were matched across the 3 task versions.

### Subjects gradually increase game difficulty

In all three task versions, the subjects tended to start by sampling easier games and increase game difficulty as the session progressed. This is shown in Figure 3, which shows the dot speed (Figure 3A) and %correct (Figure 3B) averaged across subjects in a sliding window of 2 consecutive games. In the 7-game version the subjects' initial exploration was random, as expected given that dot speeds were randomly organized in the choice display (Figure 3A, top colormap, games ~1–10); however, in the 64 and 64N versions the subjects showed a clear bias to begin sampling at the easiest levels located at the top left corner of the choice arrays and gradually progressed to more difficult games (Figure 3A, middle and lower colormaps). Linear regression on the average speed of the selected games (Figure 3A, bottom panel) showed a significant increase for the 7-game, 64-game, and 64N-game versions (with slope coefficients of, respectively, 0.053°/s/game (standard error (SE) 0.0089, *p* < 10^−5^), 0.193°/s/game (SE 0.019, *p* < 10^−10^) and 0.578°/s/game (SE 0.042, *p* < 10^−14^). In parallel, performance significantly decreased with time for all three conditions (Figure 3B; linear regression for the 7-game, 64-game, and 64N versions gave slopes of, respectively, −0.093°/s/game (SE 0.027, *p* < 0.02), −0.323°/s/game (SE 0.050, *p* < 10^−6^) and −0.402°/s/game (SE 0.03, *p* < 10^−13^; all values in units of %points/game).

### Subjects sample all the available tasks

In addition to gradually increasing game speed, the subjects tended to sample the entire range of the available tasks (colormaps in Figure 3A). Consistent with this trend, the overall distribution of game speeds across the session in the 64-game condition showed a long tail and a significantly higher mean relative to the 7-game condition (Figure 4A; means and s.e.m. of 25.63 ± 0.57°/s vs. 19.86 ± 0.24°/s, *p* < 10^−18^, One-Way ANOVA). Moreover, subjects tended to sample the most difficult games: each subject did so in the 7-game version, and a majority did so even in the 64-game and 64-N game versions (Figure 4B) where the games were extremely difficult and could not be mastered (~10% correct in Figure 2A). Thus, subjects seemed motivated to discover the entire range of the available tasks.

The availability of novel sequences produced a further increase in speed, suggesting that novelty provided an additional impetus for exploration. Game speed was significantly higher in the 64N vs. the 64-game condition across the entire session (Figure 4A; average speed (and s.e.m.) was 29.48 ± 0.63°/s vs. 25.63 ± 0.57°/s, *p* < 10^−4^, One-Way ANOVA). As shown in Figure 3A, this difference was especially pronounced during the last 20 games, when the subjects showed a large increase in speed (36.37 ± 1.24°/s vs. 29.54 ± 1.23°/s, *p* < 10^−3^, One-Way ANOVA) and a significantly lower performance in the 64N relative to the 64 version (41 ± 0.06%correct vs. 50 ± 0.06%correct, *p* < 10^−3^, One-Way ANOVA). Thus, especially toward the end of the sessions, the availability of novel sequences motivated subjects to sample more difficult games.

### Subjects show a pronounced preference to repeat games

To further understand the subjects' behavior, we examined whether their choices may be influenced by a local strategy—i.e., whether they tended to increase, decrease or repeat game difficulty according to their performance on the preceding game (Figure 5). We divided each subject's games into 6 performance ranges and for each game computed the likelihood that the subject will increase, decrease or repeat difficulty level on the next game (see Methods for details). To estimate how this analysis is constrained by the bounds of the choice set, we simulated the performance of a set of virtual subjects who were matched for performance with the real subjects but had a random choice strategy (see Methods for details).

As shown in Figure 5, under a random selection strategy, there is a consistent relationship between %correct on the current trial and the likelihood of increasing/decreasing difficulty on the next trial, which is trivially imposed by the limits of the choice sets. If the subjects are already playing a difficult game (low %correct) they tend to reduce rather than increase difficulty on the next trial (left vs. middle columns) simply because they run out of options for more difficult games. Similarly if the subjects are already playing an easy game (high %correct) they tend to increase rather than decrease difficulty on the next trial (left vs. middle columns) simply because they run out of options for easier games. As expected, the subjects' performance also showed these basic trends (Figure 5, solid colored traces), but their performance differed from a random strategy in two important ways. First, the subjects showed a lower tendency to change (decrease or increase) game difficulty than was predicted by chance (left and middle columns), and a much higher likelihood to *repeat* a level (right column). Second, while a random strategy predicts a uniform distribution of repetition rates, the subjects' repetitions were biased toward more challenging games.

To further examine this result we tested how the subjects' repetition rates interacted with their time-dependent choice strategy (Figure 3) by calculating the fraction of repetitions in the first, second and last 1/3 of the games in a session. The repetition pattern changed during the course of a session and was dependent on the choice set (Figure 6). In the first 1/3 of the games in a session, the repetition rate showed an inverse-U function that peaked for intermediate difficulty games regardless of the choice set (Figure 6A, top). A Two-Way ANOVA on these early games showed a significant effect of performance (*p* < 0.0023) but no effect of task version (*p* = 0.312 for main effect, *p* = 0.831 for interaction).

However, during the second and last thirds of the games, the repetition pattern became dependent on the choice set (*p* < 0.0013 for effect of task version, *p* = 0.777 for effect of performance, *p* = 0.45 for interaction; Two-Way ANOVA on the pooled data). One important factor driving this effect was the fact that repetition rates were biased toward the most difficult games in the 7-game version but not in the 64-game and 64N versions. Linear regression of repeat probability vs. performance gave slopes of −0.274 for the 7-game version (SE 0.139, *p* = 0.053), vs. only 0.249 (SE 0.154, *p* = 0.112) for the 64-game version and 0.139 (SE 0.145, *p* = 0.343) for the 64N version. The bias in the 7-game version can also be appreciated from the top colormap in Figure 3A, which shows a focus on the most difficult games toward the end of the session, and from the fact that, while the mean of the speed distribution was lower in the 7-game relative to the 64-game version, the *mode* of the distribution was higher (Figure 4A, modes of, respectively, 25°/s vs. 17°/s). Thus, subjects tended to repeat more difficult games, especially late in the session and in the smaller task set.

A second factor affecting repetition rate was novelty, as subjects had overall higher repetition rates in the 64N relative to the 64-game conditions (Two-Way ANOVA, *p* = 0.066 for task, *p* = 0.200 for % correct, *p* = 0.47 for interaction). This effect seemed most apparent for games of moderate and high difficulty (Figure 6A) consistent with the overall increase in speed in the 64N relative to 64-game condition (Figure 4A). Importantly however, the subjects requested novel sequences selectively according to performance: within the set of repeated games, they preferred novel sequences for the easier games, but tended to repeat the same exact sequence for more difficult games (Figure 6B; Two-Way ANOVA revealed a significant interaction between performance and repetition type (*p* = 0.0479) with no main effects of the two factors (respectively, *p* = 1.0 and *p* = 0.496). Therefore, the subjects were not automatically attracted to novel sequences, but requested them strategically when they had mastered a game.

### Monitoring of learning progress

A strategy that was found to be effective for guiding robotic exploration is based on the maximization of learning progress, whereby robots monitor their own learning and focus on tasks where their abilities most rapidly improve (Oudeyer et al., 2007; Mirolli and Baldassarre, 2013). To see whether we could find evidence for such a strategy in the present task, for the subjects that played the 64-game version we selected a subset of the played games and asked the subjects, at the end of the session, to rate their subjective estimate of how much they had progressed in a game and how much they expect to progress in the future.

As shown in Figure 7A, the objective learning rates in the task were low, as the subjects showed small increases or even decreases in performance over repeated games. Within this limited range we found no correlation between the subjective estimates of improvement and the true rates of improvement (Figure 7A; *r* = 0.10, *p* = 0.43). Similarly, there was no correlation between the subjects' estimates and their tendency to choose a particular game (Figure 7B; *r* = −0.06, *p* = 0.14). However, the subjects' subjective sense of improvement was highly correlated with their average performance on the set of games (Figure 7C; *r* = 0.58, *p* < 10^−8^). A similar pattern was found for the estimates of *future* improvement, which were not correlated with the actual improvement (*r* = −0.00, *p* = 0.07) or with choices (*r* = −0.05, *p* = 0.26), but showed a significant correlation with the average fraction correct (*r* = 0.31, *p* = 0.003). Thus, at least under the current task conditions, subjects did not seem to have a robust sense of their rate of improvement and instead used a heuristic based on average success.

## Discussion

We have shown that, even in the absence of explicit instructions or primary rewards, human subjects spontaneously organize their exploration in a consistent fashion based on several factors. First, subjects are sensitive to task difficulty and gradually progress from easier to more difficult tasks. At the same time, however, they are sensitive to the size of the choice set and tend to sample the most difficult tasks even if these tasks far exceed their skills. Second, subjects repeat tasks much more often than predicted by chance, suggesting a tendency to practice. However, at the same time they are sensitive to novelty, reaching higher speeds and show higher repetition rates if they can request novel games. These results are consistent with theoretical and behavioral investigations suggesting that intrinsically motivated exploration is shaped by a number of factors, which may sometimes have opposing effects and are integrated with different relative weights to achieve efficient exploration in a range of contexts (e.g., Oudeyer and Kaplan, 2007; Düzel et al., 2010; Kidd et al., 2012; Gottlieb et al., 2013, Mirolli and Baldassarre, 2013; Foley et al., 2014; Taffoni et al., 2014).

Our results also suggest that the subjects used these heuristics to serve two fundamental goals: maximize their knowledge of the task space and maximize their competence across that space, consistent with the computational distinction between knowledge-based and competence-based intrinsic motivations (e.g., Oudeyer and Kaplan, 2007; Mirolli and Baldassarre, 2013). The role of knowledge-based motivation is suggested by the fact that the subjects sampled novel sequences and explored even the most difficult tasks in the 64-game versions (which they could not have mastered). The role of competence-based motivation is suggested by the fact that the subjects tended to repeat challenging games and requested novel sequences only if they performed well, suggesting an inclination to practice. In other words, subjects combined several heuristics– sensitivity to difficulty, novelty and the size of the search space—to understand the limits of the available choice set and to maximize their competence across the entire set.

Among the more sophisticated algorithms that have been proposed to guide artificial exploration are those motivated by learning progress, whereby a robot tracks its learning—change in competence—over time and uses this measure as an intrinsic reward to motivate sustained engagement with specific tasks (Oudeyer et al., 2007; Gottlieb et al., 2013). While the present experiment did not seek directly to confirm or refute this idea, the results highlight some of the difficulties that may be associated with such a strategy in biological organisms. We found that the subjects had very poor subjective estimates of their rates of progress, and instead reported heuristics based on their success rates (i.e., performance, rather than its temporal derivative). This finding most likely reflects the type of learning that we tapped into here—sensorimotor learning that was implicit and slow over the course of the session—and is consistent with a large body of literature showing that meta-cognitive reports are unreliable under such conditions (Efklides, 2006; Fleming et al., 2012a,b; Maniscalco and Lau, 2012). Therefore, exploratory mechanisms based on the monitoring of learning progress may be most beneficial conditions where the learning is fast, explicit or differs clearly across tasks, consistent with computational models showing that the efficiency of different exploration strategies differs across tasks (Lopes and Oudeyer, 2012).

In sum, our study describes features of the intrinsically motivated exploratory strategies used by adult humans in a free-play context, and sets the stage for future studies testing to what extent these strategies generalize to different conditions and what are their underlying mechanisms.

### Conflict of interest statement

The authors declare that the research was conducted in the absence of any commercial or financial relationships that could be construed as a potential conflict of interest.
